# High Familial Recurrence of Congenital Heart Defects in Laterality Defects Patients: An Evaluation of 184 Families

**DOI:** 10.1007/s00246-021-02656-4

**Published:** 2021-06-19

**Authors:** Huifang Hu, Weicheng Chen, Wei Sheng, Guoying Huang

**Affiliations:** 1 Children’s Hospital of Fudan University, Institutes of Biomedical Sciences, Fudan University, Shanghai, 201102 China; 2 Cardiovascular Center, Children’s Hospital of Fudan University, No. 399 Wanyuan Road, Shanghai, 201102 People’s Republic of China; 3 Institute of Pediatrics, Shanghai Institute for Pediatric Research and Key Laboratory of Birth Defects, Shanghai, 201102 People’s Republic of China

**Keywords:** Congenital heart defects, Laterality defects, Recurrence risk, Genetic counseling, Birth defects

## Abstract

As a rare disease with genetic pathogenesis, observational study about familial CHD recurrence risk on CHD patients with laterality defects is lacking. This study aimed to investigate familial recurrence among families of patients with CHD and laterality defects, and compare them with CHD patients without laterality defects. A total of 184 patients with CHD and laterality defects treated in Cardiovascular Center, Children’s Hospital of Fudan University were observed from 2008 to 2019. A detailed family history was documented by trained staff using questionnaires, and information about the subtypes of CHD and laterality defects was also collected. In addition, positive family history information, including all three degrees relatives and all affected family members, was reconfirmed by trained medical staff through face-to-face interviews, telephone interviews, and letter return visits. Of the 184 included patients, 30 had at least one family member (from among three linear generations and distant relatives) with CHD. The familial recurrence rate of CHD in our cohort was 16.3% (30/184), which was higher than the 3.3% (67/2024) of patients with CHD without laterality defects. This result shows that the recurrence rate among the first-, second-, and third-degree relatives was 11.7% (11/94), 1.5% (3/204), and 3.1% (6/91) and that the recurrence rate among siblings (21.4%, 9/42) was higher than that among parents (3.8%, 2/52). The familial recurrence risk of CHD among patients with CHD and laterality defects is high, which is consistent with the previous study that reported a high familial recurrence of heterotaxy of 10%. First-degree relatives have a higher recurrence rate than second- and third-degree relatives, especially siblings. These findings have important significance for prenatal screening, intervention, and genetic counseling in the Chinese population, but may not be generalizable to other populations that may have different rates of familial and sporadic cases.

**Supplementary Information:**

The online version contains supplementary material available at 10.1007/s00246-021-02656-4.

## Introduction

Congenital heart disease (CHD) is considered the most common congenital disease, accounting for approximately one-third of all birth defects in newborns [1], and is the most common cause of neonatal death [2, 3]; the prevalence of neonatal CHD in China ranges from 0.89% to 2.66% [1, 4, 5]. As a disease that is highly suggestive of genetic factors, familial cases of CHD have been reported in nearly all types of cardiac malformations [1, 4]. Existing reports show that the familial recurrence rate of CHD in the population of children with CHD ranges from 3 to 5% worldwide [6]. However, when classified by subtype, the familial CHD recurrence risk ratios differ greatly and range from threefold to 80-fold compared with the prevalence in the general population [7]. Moreover, a study on the recurrence of CHD among siblings showed a similar result [8]. The sibling recurrence risk ratio of CHD varies when categorized by different CHD phenotypes. To a certain extent, these differences in familial recurrence risk ratios may reflect the different pathogeneses between different phenotypes. Clinically, more in-depth research about familial recurrence CHD is of great significance for providing more accurate genetic counseling. When families that have existing patients with CHD want to have another child, prenatal consultation is necessary.

Although the body is left–right symmetrical on the exterior, the internal organs are arranged left–right asymmetrically [9, 10], a basic feature of vertebrates. During embryonic development, left–right axis developmental abnormalities will result in abnormal internal organ arrangement in the thoracic and abdominal cavities and are usually defined as laterality defects; this phenotype ranges from isolated dextrocardia or situs inversus (SI) abdominis to situs inversus totalis (SIT), or heterotaxy (HTX) [11].

Laterality defects are rare multiple congenital malformations. The prevalence of laterality defects is approximately one per 10,000–20,000 live births [11]. The heart is a highly asymmetrical organ, and 82.8% of patients with HTX and 26.6% of patients with SIT have complex CHD [11], which leads to severe morbidity and mortality [12]. In patients with CHD and laterality defects, there may be a more specific, intrinsic pathogenesis, which deserves further study.

Notably, a past study reported that the familial recurrence risk ratio in patients with HTX or congenitally corrected transposition of the great arteries (ccTGA) is significantly higher than that in patients with other CHD phenotypes [12]. However, due to the low prevalence, the number of patients with CHD and heterotaxy included in past studies was not more than 100 in total [8, 13].

Moreover, there are a number of studies on hereditary CHD patients with heterotaxy, which indicate the genetic characteristics of this disease [14–18]. However, no observational study of the familial recurrence risk of these patients with laterality defects has been conducted. Previous research was carried out more than 10 years ago [19]. The study noted that the familial recurrence rate of heterotaxy is 10% [19], which is higher than that of other CHDs. Moreover, it may not be accurate to refer to data published more than ten years ago [19]. Differences in the population and research methods can also lead to bias. Familial recurrence research is indispensable for clinical genetic counseling and further exploration of the pathogenesis. The aim of our study was to investigate the familial recurrence risk of CHD in patients with all types of CHD and laterality defects to provide a reference for consultation in clinical practice in a Chinese population.

## Methods

### Population

From January 2008 to August 2019, patients with CHD and laterality defects who were treated at the Children’s Hospital of Fudan University, Shanghai, China, were enrolled in our study. A detailed family history was documented by trained staff using questionnaires. In addition, positive family history information, including three degrees of relatives of all enrolled patients, was reconfirmed by trained medical staff through face-to-face interviews, telephone interviews, and letter return visits. Based on the questionnaires, the parents of the proband were first interviewed. Furthermore, the immediate family members (children or parents) of affected relatives were interviewed to confirm the phenotype. We defined first-degree relatives as parents or siblings; second-degree relatives as grandparents or uncles/aunts; third-degree relatives as 1st cousins of probands; and distant relatives as relatives beyond these three linear generations. We defined the probands who had at least one family member with CHD as family history-positive cases; otherwise, they were considered family history-negative cases. The present study was approved by the local Ethics Committee of Fudan University (Shanghai, China). Written informed consent was obtained from the guardians of the probands and their family members.

### Phenotype Ascertainment

We reviewed all available medical records of the probands to confirm phenotypes (including laterality defects, CHD and congenital malformations other than CHD and laterality defects), including abdominal ultrasound, chest radiography, and/or surgical records. For the family members involved, we confirmed phenotypes mainly by reviewing their existing medical records both from our hospital and other hospitals, and having them undergo clinical examination.

### Definitions and Inclusion Criteria

In this study, we defined laterality defects as any abnormal arrangement of thoracic and abdominal viscera, including that of the heart, stomach, *liver*, spleen, gastrointestinal tract, and lung. Patients with CHD were considered to have abnormalities in veins, atria and ventricles, ventricular outflow, and great vessels based on the classification used by Botto et al. [20] and Øyen et al. [21]. In addition, we also focused on congenital malformations other than CHD and laterality defects. We also noted patients with the syndrome.

### Statistical Analysis

We calculated the familial recurrence of all involved probands, and based on their ancestral information, the recurrence of CHD among three degrees of relatives for the first-, second- and third-degree generations was calculated among all three degrees of relatives (94 first-degree relatives, 204 second-degree relatives, and 191 third-degree relatives) from 30 positive history families; the 95% confidence interval (CI) was also reported. We also calculated the prevalence of CHD and the laterality defect phenotype among probands and affected family members, and compared the prevalence of CHD and laterality between probands from two different groups of probands (positive family history cases vs. negative family history case). The data were analyzed using GraphPad Prism (version 7.0). The associations between the two groups were analyzed using Pearson’s chi-squared tests or Fisher’s exact tests, and p < 0.05 was considered statistically significant.

## Results

### Demographic Characteristics of the Probands

From January 2008 to August 2019, 357 patients with CHD and laterality defects were diagnosed at our hospital. In 184 of these patients (106 males and 78 females), the age at the time of recruitment was 8.5  ±  46.3 months (the age is presented as the mean  ±  standard deviation (SD); median = 8 months, range from 0 to 241 months). We obtained family history information by questionnaire.

### Phenotypic Statistics of Probands

Among the 184 collected probands, we conducted an analysis of CHD with laterality defects phenotype. Based on all available records, 64.1% of patients presented an abnormal heart position. Among the abdominal organs of all probands, abnormal position or absence was seen for the stomach (44.6%), the liver (56.0%), the lung (28.8%), the gastrointestinal tract (3.3%), and the spleen (62.0%). For the 30 probands with positive family history, abnormal position or absence was seen for the heart (60.0%), the stomach (50.0%), the liver (66.7%), the lung (30.0%), the gastrointestinal tract (3.3%), and the spleen (66.6%) (Table 1). In addition, the organ arrangement of each proband with a positive family history is described in Supplemental Table 2.

**Table 1:** Organ situs and CHD phenotype

Organ situs arrangements	Positive case^a^	Negative case^b^	P*-value	Total case
	(*n* = 30)	(*n* = 154)		(*n* = 184)
	n (%)	n (%)		n (%)
Heart				
Normal	12 (40.0)	54 (35.1)	0.6061	66 (35.9)
Opposite	18 (60.0)	93 (60.4)	0.9682	111 (60.3)
Midline	0 (0.0)	7 (4.5)	0.2338	7 (3.8)
No record	0 (0.0)	0 (0)	–	0 (0.0)
Stomach				
Normal	10 (33.3)	52 (33.8)	0.9634	62 (33.7)
Opposite	15 (50.0)	67 (43.5)	0.5127	82 (44.6)
No record	5 (16.7)	35 (22.7)	0.4616	40 (21.7)
Liver				
Normal	4 (13.3)	40 (26)	0.1376	44 (23.9)
Opposite	15 (50.0)	56 (36.4)	0.1604	71 (38.6)
Midline	5 (16.7)	27 (17.5)	0.9089	32 (17.4)
No record	6 (20.0)	31 (20.1)	0.987	37 (20.1)
Spleen				
Normal	3 (10.0)	28 (18.2)	0.2734	31 (16.8)
Right-sided	13 (43.3)	50 (32.5)	0.2512	63 (34.2)
Asplenia	6 (20.0)	36 (23.4)	0.6869	42 (22.8)
Polysplenia	1 (3.3)	8 (5.2)	0.6654	9 (4.9)
No record	7 (23.3)	32 (20.8)	0.7542	39 (21.2)
Gastrointestinal tract				
Normal	9 (30.0)	26 (16.9)	0.094	35 (19.0)
Biliary Atresia	0 (0.0)	4 (2.6)	0.3721	4 (2.2)
Malrotation	1 (3.3)	1 (0.6)	0.1946	2 (1.1)
No record	20 (66.7)	123 (79.9)	0.1119	143 (77.7)
Lung				
Normal	5 (16.7)	19 (12.3)	0.5195	24 (13.0)
Inverted	6 (20.0)	19 (12.3)	0.2625	25 (13.6)
Left Isomerism	1 (3.3)	6 (3.9)	0.8828	7 (3.8)
Right Isomerism	1 (3.3)	18 (11.7)	0.1689	19 (10.3)
Lung dysplasia	1 (3.3)	1 (0.6)	0.1946	2 (1.1)
No record	16 (53.3)	91 (59.1)	0.5587	107 (58.2)
CHD phenotypes				
Venous anomalies				
Interrupted inferior vena cava	2 (6.7)	17 (11.0)	0.4715	19 (10.3)
Bilateral superior vena cava	7 (23.3)	28 (18.2)	0.5107	35 (19.0)
Anomalous pulmonary venous return	4 (13.3)	21 (13.6)	0.9647	25 (13.6)
Atria and ventricles				
Atrioventricular discordance	8 (26.7)	45 (29.2)	0.7775	53 (28.8)
Atrioventricular septal defect	7 (23.3)	42 (27.3)	0.6552	49 (26.6)
Single-atrium morphology	1 (3.3)	28 (18.2)	**0.0412**	29 (15.8)
Single-ventricle morphology	10 (33.3)	49 (31.8)	0.8708	59 (32.1)
Atrial septal defect	15 (50.0)	74 (48.1)^d^	0.8451	89 (48.4)
Ventricular septal defect	12 (43.3)	51 (33.1)^e^	0.2824	64 (34.8)
Atrioventricular valve stenosis/atresia	5 (16.7)	26 (16.9)	0.9769	31 (16.8)
Ventricular outflow and great vessels				
Transposition of the great artery	15 (50.0)	74 (48.1)	0.8451	89 (48.4)
Double-outlet right ventricle	10 (33.3)	39 (25.3)	0.3639	49 (26.6)
Pulmonary stenosis	12 (40.0)	47 (30.5)	0.3088	59 (32.1)
Pulmonary atresia	7 (23.3)	29 (18.8)	0.5696	36 (19.6)
Aortic stenosis	0 (0.0)	1 (0.6)	0.6581	1 (0.5)
Coarctation of the aorta	0 (0.0)	2 (1.3)	0.5303	2 (1.1)
Right aortic arch^c^	2 (6.7)	6 (3.9)	0.496	8 (4.3)
Positive case^a^	–	–	–	30 (16.3)

*Positive case vs. negative case

^a^Proband with positive CHD familial history

^b^Proband without positive CHD familial history

^c^Exclusion of patients with dextrocardia

^d^8 of 74 probands show isolated atrial septal defect (ASD)

^e^5 of 51 probands show isolated ventricular septal defect (VSD)

In the CHD phenotype, the most prevalent phenotype was transposition of the great artery (TGA) and atrial septal defect (ASD), both accounting for 48.4% of these patients. This was followed by ventricular septal defect (VSD), which accounted for 34.8% of these patients. Of 184 probands, 13 showed isolated CHD (8 ASD and 5 VSD). All 13 probands were familial negative cases (Table 1).

### Analysis of the Familial Recurrence Rate of CHD

Of 184 participants, 30 had at least one family member with CHD. The familial recurrence rate of CHD among our cases was approximately 16.3% (Table 1). The sex distribution among the 30 patients (13 females, 17 males) with a positive family history and 154 patients (65 females, 89 males) without a positive family history showed no significant difference (P = 0.9091). In another study, we used the same methodology where we successfully reviewed 2024 discontinuous adoptions of patients with CHD without laterality defects diagnosed at our hospital during the same period. Approximately 67 patients with a positive family history were found, as indicated by statistics showing that the familial recurrence rate was approximately 3.3% (unpublished research content).

In addition, among the 46 enrolled affected family members (11 first-degree, 3 second-degree, 6 third-degree, and 26 distant relatives) from the 30 families with positive family history, 20 belonged to three linear generations, and VSD was observed among the highest number of affected family members (13 affected family members), followed by ASD and tetralogy of Fallot (TOF) (three and four affected family members, respectively). In addition, 10 affected family members died of unspecified lethal CHD when they were young, but we failed to obtain the specific phenotype (Table 2). Moreover, almost all positive families showed discordance of the CHD phenotype. Only one family showed the concordance phenotype of ASD (Supplemental Table 1), but for affected family members with an unspecified phenotype, the situation was unknown. For those family members with an unspecific phenotype, we confirmed that they had CHD through the diagnosis experience described by patients’ parents or children. Due to the long period, detailed medical diagnostic data were lost, and the accurate phenotypes of these patients could not be determined.

**Table 2:** Recurrence of different CHD subtypes in the affected family members

CHD subtypes^a^	Total cases
	(*n* = 46)
	n (%)
VSD	13 (28.3)
ASD	3 (6.5)
TOF	4 (8.7)
PDA^d^	1 (2.2)
TA/SV/ASD	1 (2.2)
VSD/PDA	1 (2.2)
Unspecified lethal CHD^b^	10 (21.7)
Unknown^c^	12 (26.1)

*VSD* ventricular septal defect, *ASD* atrial septal defect, *TOF* tetralogy of fallot, *PDA* patent ductus arteriosus, *TA* tricuspid atresia, *SV* single ventricle

^a^CHD, congenital heart defects

^b^The patients died of CHD in childhood, but the specific phenotype could not be known

^c^The patients had CHD, but the specific phenotypes were not obtained

^d^Newborn PDA is excluded

We enrolled 94 first-degree relatives, 204 second-degree relatives, and 191 third-degree relatives in order to conduct an analysis of CHD prevalence among relatives of patients with CHD and laterality defects from all 30 probands’ families with positive family history. The results showed that the prevalence in first-, second-, and third-degree relatives was 11.7%, 1.5%, and 3.1%, respectively. The prevalence among siblings (21.4%) was higher than that among parents (3.8%). The prevalence in all three degrees of relatives was 4.1% (Table 3). Only one proband was the descendant of a consanguineous marriage: the two grandmothers of the proband were sisters, but familial recurrence was not found in this family. In the remaining 183 probands, no consanguineous marriages were found.

**Table 3:** Prevalence of congenital heart defects among the three degrees of relatives of probands

Relationship to proband	Affected/Total	Prevalence (%)	Prevalence (95% CI)	P-value	
Total first-degree relatives	11/94	11.7	5.2–18.2	**0.0003**^**a**^	**0.0076**^**b**^
Parents	2/52	3.8	0–9.1	**0.0197**^**c**^	
Siblings	9/42	21.4	9.0–33.8		
Total second-degree relatives	3/204	1.5	0–3.1	0.2771^d^	
Total third-degree relatives	6/191	3.1	0.7–5.6		
Total relatives	20/489	4.1	2.3–5.8		

^a^Total first-degree relatives vs. Total second-degree relatives

^b^Total first-degree relatives vs. Total third-degree relatives

^c^Parents vs. Siblings

^d^Total second-degree relatives vs. Total third-degree relatives

### Phenotypic Statistics of Different Groups of Probands (Positive Cases Versus Negative Case)

We also compared the phenotype prevalence of CHD and laterality defects between patients with and without positive family history to explore differences in phenotype prevalence among probands with or without a family history. Eighteen main types of congenital malformations of the heart and abnormal organ situs arrangements were observed (Table 1), among which the cases with or without a family history did not differ significantly in CHD phenotypes, with the exception of single-atria morphology, which was more frequent in negative cases (Table 1). In addition, of all 489 relatives (within three degrees of relatives), 8 had VSD or ASD (7 VSD and 1 ASD) (Supplemental Table 1), and the recurrence rates were 1.43% (7/489, VSD) and 0.20% (1/489, ASD).

In addition to CHD, we also observed one proband who was diagnosed with Kaufman oculocerebrofacial syndrome by genetic testing, but the proband did not have a positive family history.

Congenital malformations other than CHD and laterality defects in family members and probands are shown in Supplement Table 4. The 1st cousin of one proband’s father had SIT, the identical twin brother of one proband presented nystagmus, and another proband’s sibling had multiple malformations. However, none of these three family members showed a cardiac abnormality (Supplemental Table 4).

## Discussion

CHD is a multifactorial disease, and both genetic and environmental factors play an important role in its development [22]. Previous studies have shown that the risk of familial recurrence in patients with CHD is higher than in the general population [7, 23], which may imply the pathogenesis of the disease. In the case of patients with CHD and laterality defects, the prompt in abnormal embryonic development may be more specific, but existing studies on patients with CHD and laterality defects are lacking. In this study, we focused on the patients with CHD and laterality defects and discovered that the recurrence rates in first-, second-, and third-degree relatives were 11.7%, 1.5%, and 3.1%, and that the recurrence rate among siblings (21.4%) was higher than that among parents (3.8%). In addition, the familial recurrence rate of CHD among these patients was 16.3%. The significantly higher familial recurrence rates than that of patients with CHD alone suggest that the pathogenesis was more evident in patients with CHD and laterality defects.

Furthermore, all known affected family members showed only the phenotype of CHD without abnormal positioning of organs. The CHD subtypes of affected family members were variable. Some patients showed a milder VSD or ASD phenotype, whereas others showed an unspecified lethal CHD phenotype, and none of these patients survived to adulthood. For those patients lacking detailed medical data, we have done our best to obtain diagnostic information by contacting the parents or children of these patients, but due to the loss of the detailed medical diagnosis, we failed to obtain accurate phenotypic information.

As CHD is a multifactorial disease, the causes of nearly 80% of cases remain unclear [22]. The different phenotypes of CHD may also be affected by many unknown factors, which may contribute to the differences in phenotypes among the affected family members.

Based on phenotypic classification studies, TGA, ASD, and VSD were the most frequently observed phenotypes, which is consistent with the previous epidemiologic studies showing that septal defects (VSD and ASD) are the most common forms of CHD, and account for approximately half of all CHD cases [22]. The recurrence rates of VSD and ASD in positive family members were 1.43% (VSD) and 0.20% (ASD), which are higher than the prevalence in Chinese live births of 0.33% (VSD) and 0.17% (ASD) [5]. The prevalence of TGA in our cohort was 48.4%, which is significantly higher than that in all CHDs (5–7%) [24]. According to previous studies, TGA is often associated with laterality defects [25], which explains why the prevalence of TGA was higher in our cohort. The comparison between patients with and without positive family history showed no significant difference, with the exception of single-atria morphology, which was more frequent in negative cases (Table 1). The distribution of CHD phenotypes among patients with CHD and laterality defects can provide more useful evidence to assess the role of genetic and environmental factors in the development of CHD.

Based on the analysis of three degrees of relatives, the recurrence rates of CHD in the first-, second-, and third-degree relatives were 11.7%, 1.5%, and 3.1%, respectively. The investigation of the familial recurrence of CHD in the nuclear family (parents and siblings) showed that the recurrence risks were 4.1% and 21.4%, respectively. The familial recurrence risk of CHD in siblings is higher than that in parents. This may be because for some serious forms of CHD, survival to adulthood and reproductive fitness are reduced, and for those parents who have a child with CHD, they may have a greater chance of giving birth to another child.

This study had several limitations. Of the 357 patients who met the phenotypic inclusion criteria, we were unable to obtain detailed family history information for 173 patients because they were lost to follow-up or refused to participate in the study, and thus, they were not included in the study, which may have caused bias. However, the phenotypic analysis of the two groups (173 uninvolved patients vs. 184 involved patients) (Supplementary Table 3) showed no significant difference. Analysis of sex differences in the two groups showed no significant difference. (173 excluded patients (67 females, 106 males) vs. 184 included patients (78 females, 106 males), P = 0.4813). Data collected in different ways (face-to-face interview, telephone interview, and letter return visits) may inevitably lead to differential misclassification. Furthermore, we cannot know the condition of siblings born outside the study period, which may result in selection bias, but it is inevitable. In addition, due to the long period, some information given to the patients from other hospitals was lost. We failed to obtain the detailed phenotypes of all affected family members, so for the affected family members with unspecified phenotypes, the situation is unknown. Furthermore, we speculate that the actual prevalence of family members may be higher. Due to limitations of CHD treatment over the past 30 years, the previous patients could not be effectively treated, and they might not have married and had offspring, which may lead to a decrease in prevalence. In addition, the lack of screening for CHD in newborns may lead to missed diagnoses. Some mild cases of CHD, such as mild VSD or ASD, may self-heal before diagnosis. Moreover, we did not perform an echocardiographic examination of all family members, so we may have missed diagnoses of CHD due to mild symptoms, both of which could lead to a decrease in the prevalence of CHD among family members. Especially in China, the birth rate is lower due to family planning, which may affect the family recurrence and phenotype distribution. Our participants’ family histories were obtained mainly through telephone consultation and review of electronic records. Inevitably, there may be deviations in the diagnosis of distant relatives, which may account for a lower familial recurrence rate than the actual situation. Moreover, as an observational study, a lack of information inevitably occurs. Although attempts were made to confirm the CHD phenotypes, there were still unspecified phenotypes of some affected family members, which is a limitation in interpreting the findings of CHD phenotypes among the relatives. Moreover, we failed to collect all positive family pedigrees, which may lead to inaccurate statistics of the recurrence rate of family members. In addition, the findings of this study are for families with laterality and CHD in China, and may not be generalizable.

This study reports that the familial recurrence risk of CHD in patients with CHD and laterality defects is higher than in those with CHD alone, and the analysis among the three degrees of relatives showed that the risk for the first-degree relatives and siblings is significantly higher than for others. Moreover, the severity of the phenotype in affected family members was polarized and showed discordance with the probands, which further proves that CHD, as a multifactorial disease, has a very complex and diverse etiology and clinical phenotype. The analysis of the phenotype and family history of patients with CHD and laterality defects gives us a deeper understanding of the disease. It also has important significance for prenatal screening, intervention, and genetic counseling. Furthermore, this family-based analysis may provide a stronger hint of genetic mechanisms, which may help in discovering potentially pathogenic genes. In recent years, many gene mutations have been confirmed to be related to abnormalities of left–right axis development in animal models, including ACVR2B, ZIC3, NODAL, LEFTY2, MMP21, SHROOM3, etc. In addition, an increasing number of cilia-related gene mutations have been confirmed to be closely related to CHD and laterality, such as *Foxj1, Anks6, Bicc1, Tbc1d32* and *Tbc1d32* [14–18], which also suggests that the disease has a genetics component. Our study is complementary to previous research, and provides a reference for further work on genetic markers in families.

## Supplementary Information

Below is the link to the electronic supplementary material.

Supplementary file1 (DOCX 32 kb)

## Data Availability

We will make all data (all tables) available upon request. The data set will be archived for at least 10 years after publication.

## Abbreviations

CHD: Congenital heart defects
SI: Situs inversus
SIT: Situs inversus totalis
HTX: Heterotaxy
ccTGA: Congenitally corrected transposition of the great arteries
CI: Confidence interval
TGA: Transposition of the great
ASD: Artery atrial septal defect
VSD: Ventricular septal defect
TOF: Tetralogy of fallot
