# Supplementary material for: High Familial Recurrence of Congenital Heart Defects in Laterality Defects Patients: An Evaluation of 184 Families
Source: Pediatr Cardiol. 2021 Jun 19;42(8):1722–9. doi: 10.1007/s00246-021-02656-4 (PMC8557144; doi:10.1007/s00246-021-02656-4)
Supplement: Supplementary file 1 — Supplementary file1 (DOCX 32 kb) [file 246_2021_2656_MOESM1_ESM.docx]

Supplemental Table 1 The phenotypes of probands and their affected family members with CHD

| Family | Proband | | |  | Family member | | | |
| --- | --- | --- | --- | --- | --- | --- | --- | --- |
|  | Gender^a^ | Phenotype^b^ | |  | Gender^a^ | Relationship^c^ | Phenotype^b^ | Concordance^d^ |
| 1 | M | SV, TGA, AVSD, RAA, VSD, PS | HTX |  | M | Father | Unspecified | U |
|  |  |  |  |  | F | Distant cousin | ASD | N |
| 2 | M | AVSD, DORV, PS, PA, ASD | HTX |  | F | Cousin | **VSD** | N |
| 3 | M | PS, ASD, AVSD, DORV, TGA | HTX |  | F | Aunt | Unspecified | U |
| 4 | M | PS, ASD, VSD | SIT |  | F | Cousin | **VSD** | N |
|  |  |  |  |  | M | Cousin | **VSD** | N |
|  |  |  |  |  | F | Distant cousin | Unspecified | U |
|  |  |  |  |  | F | Distant grandparent | Unspecified | U |
|  |  |  |  |  | M | Distant grandparent | Unspecified | U |
| 5 | M | DORV, VSD, BSVC | HTX |  | F | Sister | Unspecified lethal CHD | U |
| 6 | M | TGA, PA, VSD, ASD | HTX |  | F | Distant cousin | VSD | N |
| 7 | F | DORV, VSD, TGA, PS, BSVC | SIT |  | F | Distant cousin | ASD,SV,TA | N |
| 8 | M | SV, AVSD, TGA, PS, ASD, APVR | HTX |  | M | Distant cousin | VSD | N |
| 9 | M | SV, ASD, TGA, PS | HTX |  | F | Sister | Unspecified lethal CHD | U |
|  |  |  |  |  | F | Sister | Unspecified lethal CHD | U |
| 10 | F | ASD, DORV, BSVC, SV | HTX |  | F | Distant cousin | VSD | N |
|  |  |  |  |  | U | Distant cousin | Unspecified lethal CHD | U |
| 11 | F | VSD, ASD, APVR | HTX |  | M | Uncle | **VSD** | N |
| 12 | F | PS, DORV, SV | SIT |  | M | Cousin | Unspecified lethal CHD | U |
| 13 | F | PS, PDA, ASD | HTX |  | F | Twins | PDA | N |
| 14 | F | DORV, VSD, APVR, PDA | HTX |  | M | Uncle | **ASD** | N |
|  |  |  |  |  | F | Distant aunt | VSD | N |
|  |  |  |  |  | F | Distant grandparent | VSD | N |
|  |  |  |  |  | F | Distant grandparent | ASD | N |
| 15 | M | RAA, DORV, BSVC | SIT |  | F | Distant grandparent | Unspecified | U |
|  |  |  |  |  | M | Cousin | **VSD** | N |
|  |  |  |  |  | M | Uncle | TOF | N |
| 16 | M | TGA | HTX |  | F | Distant cousin | TOF | N |
|  |  |  |  |  | M | Distant grandparent | Unspecified | U |
| 17 | M | SV, PA, TGA, ASD, RAA, BSVC | HTX |  | M | Twins | **VSD,**PDA,PFO | N |
| 18 | M | SV, DOLV, TGA, PS, BSVC | HTX |  | F | Aunt | Unspecified lethal CHD | U |
| 19 | M | SV, D-TGA, AVSD, ASD | HTX |  | F | Grandmother | Unspecified | U |
| 20 | F | RAA, SA, SV, PA, TGA, BSVC, i-IVC | HTX |  | F | Sister | Unspecified lethal CHD | U |
| 21 | F | ASD | HTX |  | F | Twins | TOF | N |
| 22 | F | PA,VSD, ASD, TGA, APVR | HTX |  | F | Cousin | Unspecified | U |
| 23 | M | RAA,PA, AVSD, VSD | HTX |  | U | Little sibling | Unspecified lethal CHD | U |
| 24 | F | TGA, PS, ASD, DORV, VSD, SV | HTX |  | M | Distant uncle | VSD | N |
| 25 | F | AVSD, TGA, PA, RAA | HTX |  | M | Father | TOF | N |
| 26 | M | i-IVC, TGA | HTX |  | F | Cousin | Unspecified | U |
|  |  |  |  |  | F | Distant cousin | Unspecified lethal CHD | U |
|  |  |  |  |  | M | Distant uncle | Unspecified | U |
|  |  |  |  |  | M | Distant cousin | Unspecified | U |
| 27 | M | VSD, ASD | HTX |  | F | Distant aunt | VSD | N |
| 28 | F | VSD | SIT |  | M | Distant cousin | VSD | Y |
| 29 | M | DORV, PS | SIT |  | U | Little sibling | Unspecified lethal CHD | U |
| 30 | F | VSD | SIT |  | M | Brother | DORV,CoA**,VSD** | N |

^a^F, female; M, male.

^b^VSD, ventricular septal defect; ASD, atrial septal defect; APVR, anomalous pulmonary venous return; AVSD, atrioventricular septal defect; i-IVC, interrupted inferior vena cava; BSVC, bilateral superior vena cavae; SA, single-atrium morphology; SV, single-ventricle morphology; DORV, double-outlet right ventricle; PS, pulmonary stenosis; PA, pulmonary atresia; RAA, right aortic arch; PDA, patent ductus arteriosus; TOF, tetralogy of Fallot; HTX, heterotaxy ;Unspecified lethal CHD: the patients died of CHD in childhood, but the specific phenotype could not be known. Unspecified: the patients had CHD, but specified phenotypes were not obtained;Bold indicates the affected relatives with VSD or ASD within three degrees relatives(including 7VSD and 1ASD).

^C^Cousins are 1st cousins of probands;Distant relatives are relatives beyond three lineal generations;

^d^Y, concordant; N, discordant; U, unspecified

Supplemental Table 2 Detail organ situs of probands with positive familial history

| **Proband^*^** | **Organ Situs arrangements** | | | | | |
| --- | --- | --- | --- | --- | --- | --- |
|  | **Heart** | **Stomach** | **Liver** | **Spleen** | **Gastrointestinal Tract** | **Lung** |
| 1 | Opposite | Normal | Midline | Asplenia | No record | No record |
| 2 | Opposite | Normal | Normal | Normal | No record | No record |
| 3 | Opposite | Normal | Midline | Asplenia | No record | No record |
| 4 | Opposite | Opposite | Opposite | Right-Sided | No record | No record |
| 5 | Opposite | Opposite | Opposite | Right-Sided | No record | Inverted |
| 6 | Normal | Opposite | Opposite | Right-Sided | Normal | Inverted |
| 7 | Opposite | Opposite | Opposite | Right-Sided | No record | Inverted |
| 8 | Opposite | Normal | Midline | Asplenia | Normal | Right Isomerism |
| 9 | Normal | Normal | Normal | Normal | Normal | Normal |
| 10 | Normal | Normal | Normal | Normal | No record | Normal |
| 11 | Opposite | No record | Opposite | Right-Sided | No record | No record |
| 12 | Opposite | No record | Opposite | Right-Sided | No record | No record |
| 13 | Normal | No record | No record | No record | No record | No record |
| 14 | Opposite | Opposite | Opposite | Asplenia | Malrotation | Inverted |
| 15 | Opposite | Opposite | Opposite | Right-Sided | No record | No record |
| 16 | Normal | Normal | No record | No record | No record | No record |
| 17 | Opposite | Opposite | No record | No record | Normal | No record |
| 18 | Normal | Opposite | Opposite | Right-Sided | Normal | Normal |
| 19 | Normal | No record | Midline | Asplenia | No record | No record |
| 20 | Opposite | Opposite | Opposite | Right-Sided | No record | Left Isomerism |
| 21 | Opposite | Normal | No record | No record | Normal | Lung dysplasia |
| 22 | Opposite | Normal | Midline | Polysplenia | No record | Inverted |
| 23 | Normal | Opposite | Normal | Asplenia | No record | No record |
| 24 | Normal | Opposite | Opposite | Right-Sided | Normal | Normal |
| 25 | Normal | Normal | No record | No record | No record | Normal |
| 26 | Normal | Opposite | Opposite | Right-Sided | No record | No record |
| 27 | Normal | No record | No record | No record | No record | No record |
| 28 | Opposite | Opposite | Opposite | Right-Sided | Normal | No record |
| 29 | Opposite | Opposite | Opposite | No record | No record | No record |
| 30 | Opposite | Opposite | Opposite | Right-Sided | Normal | Inverted |

^*^The proband number corresponds to the family number in Supplemental Table 1.

Supplement Table 3 Organ situs and CHD Phenotype (Involved case vs. Uninvolved patients)

| **Organ Situs arrangements** | **Involved case** | **Uninvolved patients** | P*-value |
| --- | --- | --- | --- |
|  | **(n=184)** | **(n = 173)** |  |
|  | **n（%）** | **n (%)** |  |
| **Heart** |  |  |  |
| Normal | 66(35.9) | 33(19.1) | 0.0004 |
| Opposite | 111(60.3) | 135(78.0) | 0.0003 |
| Midline | 7(3.8) | 5(2.9) | 0.632 |
| No record | 0(0.0) | 0(0.0) |  |
| **Stomach** |  |  |  |
| Normal | 62(33.7) | 19(11.0) | <0.0001 |
| Opposite | 82(44.6) | 26(15.0) | <0.0001 |
| No record | 40(21.7) | 128(74.0) | <0.0001 |
| **Liver** |  |  |  |
| Normal | 44(23.9) | 28(16.2) | 0.069 |
| Opposite | 71(38.6) | 36(20.8) | 0.0002 |
| Midline | 32(17.4) | 36(20.8) | 0.4111 |
| No record | 37(20.1) | 73(42.2) | <0.0001 |
| **Spleen** |  |  |  |
| Normal | 31(16.8) | 20(11.6) | 0.1537 |
| Right-Sided | 63(34.2) | 36(20.8) | 0.0046 |
| Asplenia | 42(22.8) | 52(30.1) | 0.121 |
| Polysplenia | 9(4.9) | 4(2.3) | 0.1935 |
| No record | 39(21.2) | 61(35.3) | 0.0031 |
| **Gastrointestinal Tract** |  |  |  |
| Normal | 35(19.0) | 13(7.5) | 0.0014 |
| Biliary Atresia | 4(2.2) | 0(0.0) | 0.0511 |
| Malrotation | 2(1.1) | 1(0.6) | 0.5986 |
| No record | 143(77.7) | 159(91.9) | 0.0002 |
| **Lung** |  |  |  |
| Normal | 24(13.0) | 21(12.1) | 0.7969 |
| Inverted | 25(13.6) | 17(9.8) | 0.2704 |
| Left Isomerism | 7(3.8) | 2(1.2) | 0.1107 |
| Right Isomerism | 19(10.3) | 7(4.0) | 0.0225 |
| Lung dysplasia | 2(1.1) | 3(1.7) | 0.6031 |
| No record | 107(58.2) | 123(71.1) | 0.0107 |
| **CHD Phenotypes** |  |  |  |
|  |  |  |  |
| **Venous anomalies** |  |  |  |
| Interrupted inferior vena cava | 19(10.3) | 7(4.0) | **0.0225** |
| Bilateral superior vena cava | 35(19.0) | 30(17.3) | 0.6809 |
| Anomalous pulmonary venous return | 25(13.6) | 34(19.7) | 0.123 |
| **Atria and ventricles** |  |  |  |
| Atrioventricular discordance | 53(28.8) | 60(34.7) | 0.2328 |
| Atrioventricular septal defect | 49(26.6) | 67(38.7) | **0.0147** |
| Single-atrium morphology | 29(15.8) | 24(13.9) | 0.6161 |
| Single-ventricle morphology | 59(32.1) | 63(36.4) | 0.3864 |
| Atrial septal defect | 89(48.4) | 79(45.7) | 0.6089 |
| Ventricular septal defect | 64(34.8) | 86(49.7) | **0.0043** |
| Atrioventricular valve stenosis/atresia | 31(16.8) | 19(11.0) | 0.1105 |
| **Ventricular outflow and great vessels** |  |  |  |
| Transposition of the great artery | 89(48.4) | 84(48.6) | 0.9721 |
| Double-outlet right ventricle | 49(26.6) | 52(30.1) | 0.4724 |
| Pulmonary stenosis | 59(32.1) | 70(40.5) | 0.0988 |
| Pulmonary atresia | 36(19.6) | 42(24.3) | 0.2816 |
| Aortic stenosis | 1(0.5) | 1(0.6) | 0.9651 |
| Coarctation of the aorta | 2(1.1) | 2(1.2) | 0.9506 |
| Right aortic arch^b^ | 8(4.3) | 4(2.3) | 0.2862 |
| Positive case^a^ | 30(16.3) | — | — |

^*^Involved case vs. Uninvolved patients

^a^Proband with positive CHD familial history

^b^Exclusion of patients with dextrocardia

Supplemental Table 4 Congenital malformations other than CHD and laterality

| **Proband** | | |  | **Relatives** | | |  |
| --- | --- | --- | --- | --- | --- | --- | --- |
| **Number** | **Gender^a^** | **Phenotype** |  | **Gender^a^** | **Relationship** | **Phenotype^b^** | **Family history^c^** |
| 1 | M | - |  | M | 1st cousin of the proband’s father | SIT | N |
| 2 | M |  |  | M | twin brother | nystagmus | N |
| 3 | F |  |  | F | sibling | multiple malformations | N |
| 4 | F | Congenital rib deformity；Lumbar hernia |  | - | | | N |
| 5 | F | Congenital sternum deformity； Congenital absence of diaphragm， Umbilical hernia |  |  |  |  | N |
| 6 | M | cheilopalatognathus |  |  |  |  | N |
| 7 | M | Congenital sternum deformity; Congenital finger deformity |  |  |  |  | N |
| 8 | M | Congenital finger deformity |  |  |  |  | N |
| 9 | M | Umbilical hernia |  |  |  |  | N |
| 10 | F | Congenital anorectal atresia |  |  |  |  | N |
| 11 | F | Cheilopalatognathus |  |  |  |  | N |
| 12 | M | Congenital sternum deformity |  |  |  |  | N |
| 13 | F | Congenital sternum deformity |  |  |  |  | N |

^a^F, female; M, male.

^b^SIT,situs inversus totalis

^c^ N, familial negative case
